# Drip tectonics and the enigmatic uplift of the Central Anatolian Plateau

**DOI:** 10.1038/s41467-017-01611-3

**Published:** 2017-11-16

**Authors:** Oğuz H. Göğüş, Russell N. Pysklywec, A. M. C. Şengör, Erkan Gün

**Affiliations:** 1 Eurasia Institute of Earth Sciences, Istanbul Technical University, Maslak, 34469 Istanbul, Turkey; 2 Department of Earth Sciences, University of Toronto, Toronto, ON Canada M5S 3B1

## Abstract

Lithospheric drips have been interpreted for various regions around the globe to account for the recycling of the continental lithosphere and rapid plateau uplift. However, the validity of such hypothesis is not well documented in the context of geological, geophysical and petrological observations that are tested against geodynamical models. Here we propose that the folding of the Central Anatolian (Kırşehir) arc led to thickening of the lithosphere and onset of “dripping” of the arc root. Our geodynamic model explains the seismic data showing missing lithosphere and a remnant structure characteristic of a dripping arc root, as well as enigmatic >1 km uplift over the entire plateau, Cappadocia and Galatia volcanism at the southern and northern plateau margins since ~10 Ma, respectively. Models show that arc root removal yields initial surface subsidence that inverts >1 km of uplift as the vertical loading and crustal deformation change during drip evolution.

## Abstract (web summary)

The recycling of continental lithosphere and rapid plateau uplift is believed to be the result of lithospheric drips, but natural examples are missing. Here, the authors use geodynamic models to suggest that the folding and thickening of the Central Anatolian arc caused lithospheric dripping of the arc root.

## Introduction

The tectonic evolution of the Mediterranean involves a complex array of subduction, collision, and back-arc spreading events in relation to ongoing northward convergence of Africa towards Eurasia^1,2^. Tectonic complexity arises from the microplate kinematics in the region inferred by geodetic studies^3^ including oroclinal bending of various orogens^4,5^, and a series of postulated lithospheric removal events^6–9^, interpreted from data showing thin, hot, and active lithosphere^10,11^.

In this work, we consider a case in Central Anatolia where folding of a Tethyan arc may have caused lithospheric instability through localized thickening of the arc root. The development of this instability as a style of “drip tectonics”, in turn, may account for the uplift (>1 km) over the entire plateau and volcanism at the plateau margins since ~10 Ma in which their cause remains enigmatic.

**Fig. 1:**
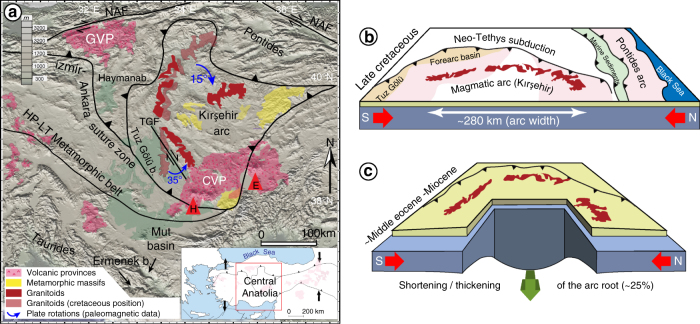
Geological setting and lithospheric evolution of Central Anatolia. **a** Generalized map of Central Anatolia showing continental blocks and their major tectonic boundaries. Simplified surface geological elements are shown to illustrate geologically inferred connection with the deep lithospheric removal process. CVP Cappadocia Volcanic Province, GVP Galatian Volcanic Province, TGB Tuz Gölü Basin, TGF Tuz Gölü Fault, NAF North Anatolian Fault, E Erciyes volcano, H Hasandağ volcano, Green area shows basins. **b** Schematic illustration of restored tectonic configuration and geologic features during the late Cretaceous^29^ showing intact lithosphere. **c** Schematic showing folding of the Central Anatolian (Kırşehir) arc about a vertical axis and ~25% shortening^31^ culminating in the Middle Eocene–Miocene^32,33^. Such folding yields local thickening of the arc root and the onset of instability

The Central Anatolian Plateau is a distinct geological region bounded by the Eastern Anatolian shortening Province to the east and the Western Anatolian Extensional Province to the west (Fig. 1a). The average topographic elevation of the low-relief plateau interior (Kırşehir block) is ~1 km, while the southern (Taurides) and northern margins (Pontides) of the plateau are defined by ~1.5 km mean elevation. River incision and paleoaltimetry studies in the Cappadocia Volcanic (south-central plateau) rocks suggest ~1 km of surface uplift since 8 Ma^12^. In the northern margin, geomorphological studies suggest >1 km of river incision in response to the surface uplift since the early Pliocene ~(5 Ma)^13^ related to the transpressional tectonics of the North Anatolian fault. Quantitative paleoelevation estimates suggest that the southern margin of Central Anatolian Plateau was below sea level during the Miocene and has experienced >2 km surface uplift since the last 8 Ma^14,15^. Furthermore, these ~8 My old sedimentary deposits in the Mut and Ermenek basins (southern margin/Taurides) (Fig. 1a) are rather undeformed and in sub-horizontal positions. Based on these and other seismological observations, the slab detachment mechanism under Cyprus has been proposed to account for the localized >2 km uplift along the southern margin of the plateau^16^. According to the cosmogenic dating of river terraces, there is surface uplift—at least since 2 Ma—across entire Central Anatolia, although the rate and magnitude in the north and south is 5 and 10 times higher than in the central section of the plateau, respectively^17^. The cause of uplift over the entire plateau—including plateau interior—remains uncertain.

Seismic tomography shows near-surface low-velocity P and S wave anomalies beneath Central Anatolia that suggest lithospheric thinning and concurrent asthenospheric mantle uprising under the crust^10,18–20^. Receiver function studies interpret that the lithosphere is only ~60 km thick under Central Anatolia^21^. The seismic tomography model^18^ indicates an attenuated piece of “V” shaped fast velocity body under the plateau interior and slow seismic anomalies at shallow depths at the northern (Pontides) and southern (Taurides) margins (N–S cross section along 33° E). Corroborating the seismological interpretations, geochemical studies from Central Anatolia volcanics emphasize the contribution of asthenospheric mantle derived magmatism. For instance, Central Anatolia extension was accompanied by the eruption of the Erciyes and Hasandağ volcanoes at the Cappadocia Volcanic Province. The Quaternary volcanics in this region are suggested to be derived from asthenospheric melts^22,23^. According to geochemical investigations from the Late Miocene Galatian Volcanic Province (in the northern margin), later stage Alkaline volcanics (~10 Ma to recent) are produced by decompression melting of the asthenospheric mantle in relation to regional extension^24–26^ (Fig. 1a).

Arc-related granitoids produced 80 Ma in the east of the Tuz Gölü basin^27^ and surrounding HP-LT rocks, metamorphosed 88 Ma^28^, suggest that the Central Anatolian (Kırşehir) block developed as a magmatic arc above an approximately eastward dipping subduction (Fig. 1a, b). Based on stratigraphic evidence^29^, the 90° rotation of the Haymana basin axis occured due to the collision of the Kırşehir block with the Central Pontides in the late Paleocene-early Eocene. This collision instigated the anticlockwise rotation of Central Anatolia in which the arc doubled back on itself by folding around a vertical axis^30^. Paleomagnetic work^31^ on the granitoids of Central Anatolia corroborated this interpretation with their estimate of a 280 km wide NNE trending plutonic belt folded into the present day position since the Cretaceous by ~25% shortening. The timing of the maximum rotation/folding is the middle Eocene–Miocene based on paleomagnetic reconstructions^32,33^.

We hypothesize that such oroclinal folding and plate shortening caused the thickening of the deep arc root lithosphere (i.e., the colder and denser part) and led to gravitational viscous instability under Central Anatolia (Fig. 1c). Reconciling model predictions with the observed tectonics, we show that lithospheric instability (dripping) model is consistent with entire surface uplift of >1 km since ~10 Ma as well as features having a symmetry in Central Anatolia, for instance, lower seismic velocities in the mantle, Galatia and Cappadocia volcanism (asthenosphere derived) in the northern and southern margins of the plateau.

## Results

### Model set-up

**Fig. 2:**
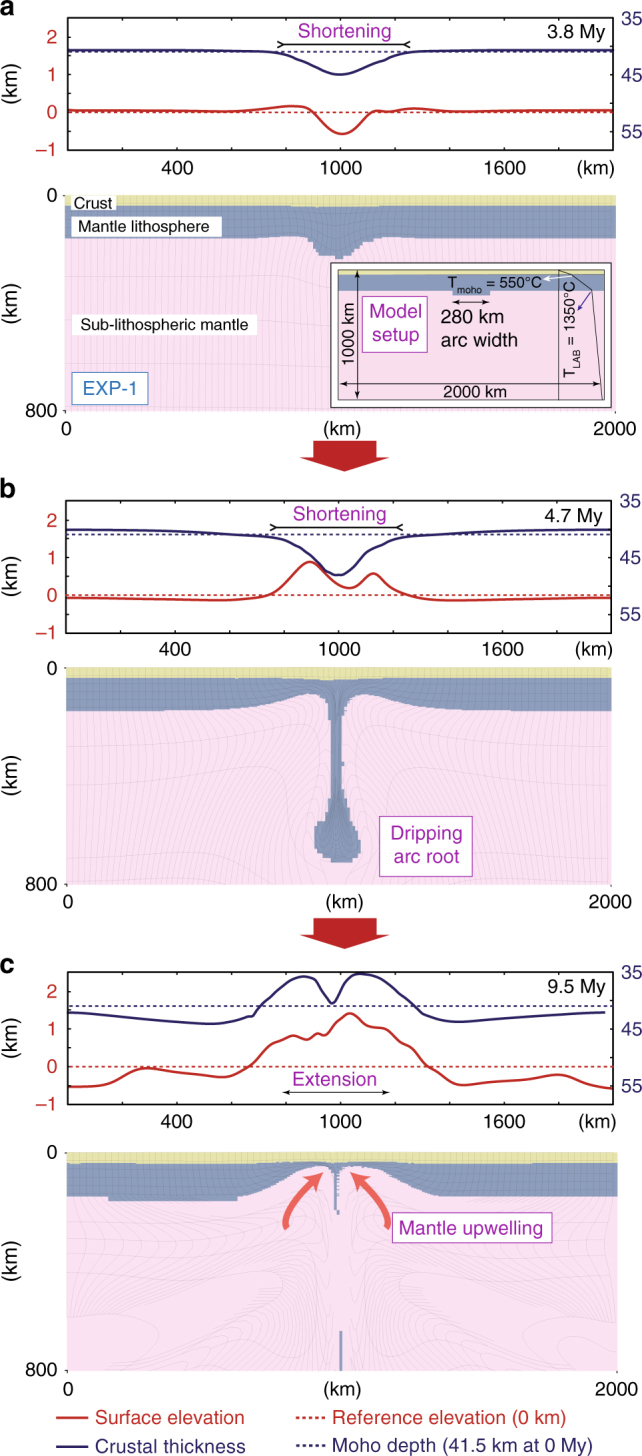
Drip tectonics model for the Central Anatolia. The geodynamic evolution of EXP-1 (preferred model) for arc root removal and the inset shows the set-up including the arc width, approximately 280 km based on paleomagnetic restorations^31^ (see Supplementary Fig. 1 for set-up description). Deforming Lagrangian mesh is overlain on material colors (yellow-crust; blue-mantle lithosphere; pink-sub-lithospheric mantle). At labeled time frames the surface topography and crustal thickness are shown. Note that the relatively minor change from the model symmetry to asymmetry is visible in the surface topography and crustal thickness variations, although not necessarily geologically relevant to Central Anatolia’s lithosphere evolution. This is due to the initial model set-up conditions in which the drip initiator at the base of the sub-arc mantle lithosphere (arc root, blue region) is shifted to the left (by several kms) from the center

Forward geodynamical models explore the dynamics and the tectonic response to the removal of ~280 km wide × 160 km thick (25% thickened), gravitationally unstable lithosphere as an approximation to the foundering of Kırşehir arc root (sub-arc mantle lithosphere) after thickening. The inset in Fig. 2a and Supplementary Fig. 1. shows the initial model set-up and the geometry of the dripping arc root lithosphere experiments. Other model parameters, such as layer thicknesses, densities and experimental parameters are described in the Supplementary Material (Supplementary Fig. 1 and Table 1). Except EXP-3, we did not impose any plate convergence from the left or right margins of the lithospheric domain (i.e., used stable boundary conditions). The description of the numerical code (SOPALE) is given in the Methods section.

In these models, density, *ρ*, is a function of composition and temperature, *ρ* = *ρ*_o_ (1–*α* (*T*−*T*_o_)), where *T* is temperature, *α* = 2 × 10^–5^ K^−1^ is the coefficient of thermal expansion, *T*_o_ = 25 °C is the reference temperature, and *ρ*_o_ is the reference density that depends on material. Experiments showed that an increase in the thermal expansion coefficient (e.g., *α* = 3 × 10^−5^ K^−1^) has relatively minor effect on these lithospheric scale model calculations.

For rheological calculations we use laboratory measurements based on a viscous flow law of $\dot \varepsilon = A\sigma^{ n} {\mathrm{exp}}\left( {\frac{{ - Q}}{{RT}}} \right)$. Here, $\dot \varepsilon$ is the strain rate, *T* is temperature, *σ* is deviatoric stress, and the variables *A*, *n*, *Q*, and *R* are the viscosity parameter, power law exponent, activation energy, and ideal gas constant, respectively. The EXP-1 (preferred model) uses the temperature-independent mantle lithosphere rheology with viscous flow law parameters *Q* = 0 and *A* = 10^−38^ Pa^−n^ s^−1^. Based on strain rates of 10^−12^ to 10^−17^ s^−1^ that are characteristic of flow in the models, this corresponds to the mantle lithosphere viscosity *μ* at the sub-arc region and elsewhere in the model ranging from 2.69 × 10^19^ to 5 × 10^23^ Pa s. For the practical purpose in these calculations we set the minimum and maximum viscosity variation ranging from 5 × 10^19^ to 5 × 10^22^ Pa s.

The initial size of the instability, based on an approximation for the width of the Kırşehir magmatic arc and other factors such as lithosphere rheology and density difference may control the propensity and nature of the instability (Supplementary Figs. 2 and 3). We show the most representative numerical models for the drip tectonics/arc root removal model in Central Anatolia from a large set of models as part of the parametric numerical study.

### Tectonic evolution of the dripping lithosphere

EXP-1 shows instability developing by 3.8 My and associated surface subsidence of ~600 m as the crust is pulled down (negative dynamic topography) by the dripping lithosphere (Fig. 2a). The crust thickens by 5 km above the drip and this corresponds to a confined zone of shortening. At 4.7 My, the surface subsidence has inverted to uplift (to 1 km) since the vertical load induced by the descending mantle lithosphere has diminished with necking of the dripping arc root (Fig. 2b). The twin-highs are raised by the upward return mantle flow at the sides of the central lithospheric downwelling. Comparing the surface topography evolution of the model with the Central Anatolian plateau, the twin-high uplift pattern on the edges of the instability may be consistent with the distinctively elevated topography in the northern and southern margins of the plateau with respect to its interior^16,17^. By 9.5 My the surface topography is characterized by a wide region of uplift with maximum elevation of 1.5 km (Fig. 2c). The descending sub-arc lithosphere has been removed and it has been replaced by hot sub-lithospheric/asthenospheric mantle. The crust has now thinned appreciably (~6 km) as a result of upwelling mantle causing extension (note that despite the crustal thinning, topographic transients are positive with an uplift). The cause of the broad uplift is a combination between the dynamic and isostatic support.

**Fig. 3:**
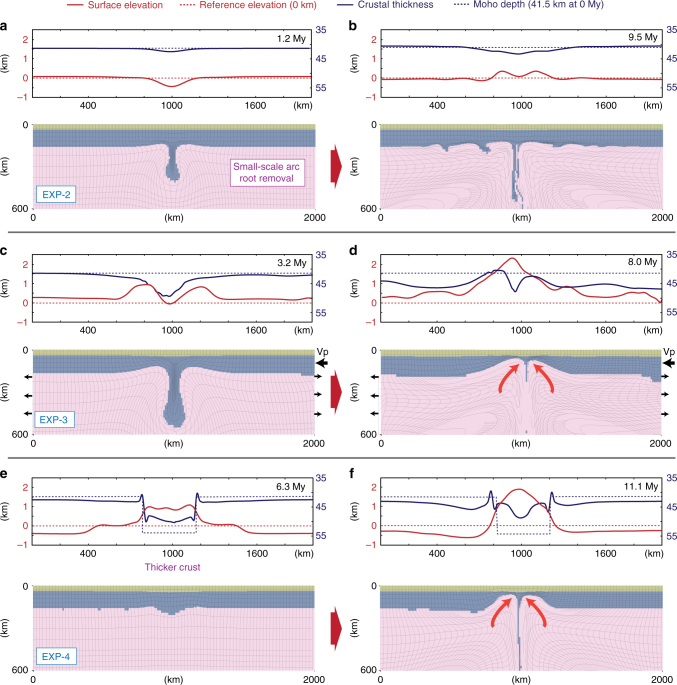
Model predictions of alternative lithospheric drip experiments. Model results as in Fig. 2 for models: **a**, **b** EXP-2 with temperature dependent and dry olivine creep law mantle lithosphere rheology^34^; **c**, **d** EXP-3 with imposed lithospheric convergence velocity of 2 cm/year, approximations to the orogenic shortening and lithospheric drip operating simultaneously^35^; **e**, **f** EXP-4 with locally increased crustal thickness of 55 km, testing the crustal response to the gravitational collapse and the lithospheric drip^37^. There is slight asymmetric development in the crustal thickness profile due to the imposed plate convergence velocity to the lithospheric domain

EXP-2 tests a similar model, but with temperature-dependent mantle lithosphere rheology (e.g., small-scale drips) based on dry olivine creep^34^ (see Methods section and Supplementary Table 1). Figure 3a shows that the localized instability grows quickly and by 1.2 My a small drip began to sink into the mantle. This dripping lithosphere yields a surface subsidence of ~500 m and crustal thickening of 2.5 km above the descending lithosphere. The surface topography evolution of this experiment is similar to EXP-1 with inversion from negative to positive topography, but the magnitude is diminished, since the amount of material participating in the downwelling process is less. At 9.5 My, a number of small-scale viscous “driplets” start to develop at the bottom of the lithosphere (Fig. 3b). The surface uplift is ~400 m and the modest crustal thickening persists. This is different from (EXP-1) where the plateau-like positive surface topography develops with large-scale removal of the arc root lithosphere. In addition, this style of removal does not lead to late-stage crustal thinning, but rather maintains thickening.

For EXP-3, a convergence velocity of 2 cm/year is imposed to consider lithospheric removal contemporaneous with shortening, such as in Cordilleran type orogenic systems^35^. All other model parameters are the same with the EXP-1. The convergence promotes faster development of the arc root instability (Fig. 3c). Shortening—driven by both the drip and the imposed convergence—yields crustal thickening of ~10 km. This causes surface uplift of ~1 km, although a trough at the center of this uplift develops as the active drip pulls down the middle of this zone. By 8.0 My, the arc root has been removed and despite the plate convergence, the mantle dynamics are sufficiently strong to permit crustal thinning above a large area of the drip zone (e.g., mainly on the margins) (Fig. 3d). There is still uplift above the localized thinned crust owing to the sub-crustal dynamics (~*x* = 800 km) (as in EXP-1). These surface deformations with convergence-enhanced convective removal are akin to the syn-convergent extension that can be induced by delamination type lithospheric removal^36^.

EXP-4 shows how post-orogenic (15 km thicker crust) modifies the evolution of the dripping lithosphere^37^ (Fig. 3e). In all other aspects, the experiment is identical to EXP-1. The model shows that an increase in the initial crustal thickness (more buoyant) has a retarding effect on the development of the lithospheric instability and the removal process. Specifically, the sinking of the gravitationally unstable arc root begins after the thinning of the 55 km thick and buoyant orogenic crust which may develop in conjunction with the post-orogenic gravitational collapse. The experiment shows that there is ~1 km surface elevation at 6.3 My and at 11.1 My, the drip has detached after removal of a large portion of the lithosphere (Fig. 3f). This results in a further uplift of surface topography, reaching 2 km. The crust, however, has thinned >10 km on the margins of the lithospheric drip. Overall, the dynamics of the lithospheric drip and its transient surface displacements develop similarly with the EXP-1 where the surface elevation increases contemporaneous to the crustal extension above the zone of the instability.

## Discussion

**Fig. 4:**
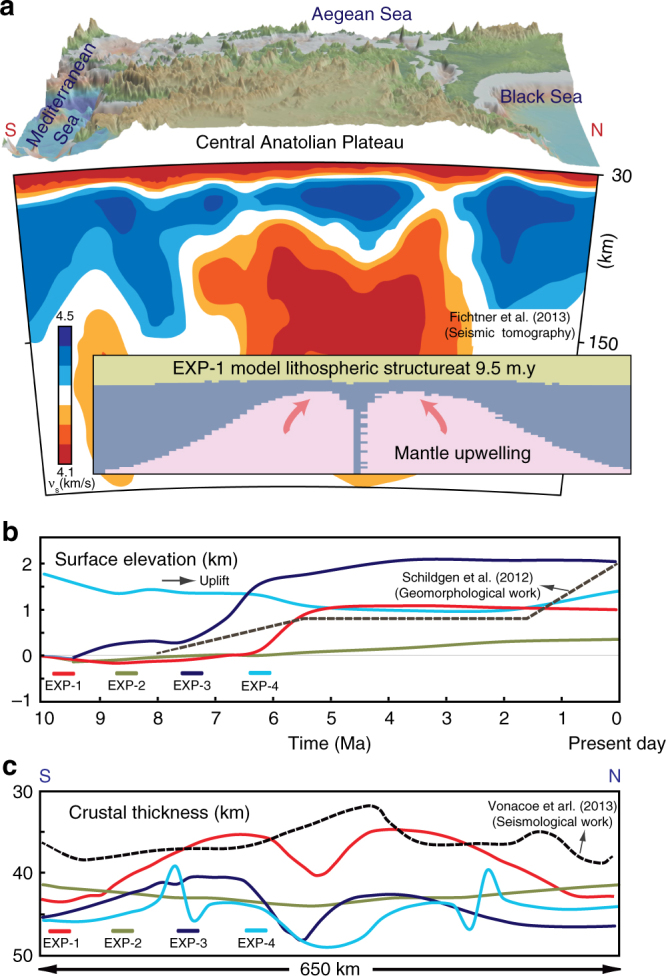
Reconciling model results against the tectonics of Central Anatolia. **a** Present day surface topography at Central Anatolia and simplified S wave tomography model along 33° E^18^. The tomography model is reconciled with EXP-1 lithospheric structure model prediction at 9.5 My (forward model time, corresponds to approximately present). **b** Time series of the modeled surface elevation for EXP-1, EXP-2, EXP-3, and EXP-4 at *x* = 850 (on the margin of the instability), comparing with: (1) estimated uplift in the Taurides)^15^ (southern margin of the plateau). **c** Crustal thickness variation along the arc root removal zone for models EXP-1, EXP-2, EXP-3, and EXP-4 (~650 km) compared against crustal thickness inferences over Central Anatolia inferred by receiver function studies^39^

Our arc root removal models of lithospheric replacement with hot sub-lithospheric mantle (e.g., EXP-1, 3, 4) are consistent with proposed seismic tomography models that show slow seismic velocities beneath Central Anatolia^10,18–21^. In particular, the symmetric features in the tomographic N–S cross section^18^ (along 33° E) (i.e., slow anomalies around the “V” shaped fast velocity body) reconcile with the predicted lithospheric structure in EXP-1 at 9.5 My (Fig. 4a). Similarly, the presence of slow seismic velocity anomalies at shallow lithospheric levels around the high velocity anomalies (Kırşehir arc) is also represented in the high resolution seismic cross section cutting through Western-Central and the Central-East Anatolia (D–D′)^18^. Geodynamic predictions also suggest that mantle upwelling—compatible with the presence of slow seismic velocity anomalies in the south and north of Kırşehir (central) block—are well correlated with the Erciyes^22^ and Hasandağ^23^ basalts of the Cappadocia volcanics and the Galatia volcanics^24–26^, (both originated from asthenospheric mantle source), respectively.

Figure 4b plots a time series of the modeled surface elevation at *x* = 850 (~150 km south of the center of instability) in all experiments compared against the inferences of estimated uplift in the southern margin of the plateau (Taurides) from ref. ^15^. In EXP-1, an inversion from subsidence to uplift occurs 8 Ma with final uplift of ~1 km, ~6 Ma (Fig. 4b). In EXP-2 with small-scale removal model, the amount of uplift is less than 500 m over 9.5 My In EXP-3 where plate convergence is imposed, the uplift is amplified compared to EXP-1 (~2 km). EXP-4, with initially thicker crust, shows surface subsidence until 2 Ma and then the elevation increases by ~500 m. Geomorphological interpretations^15^ suggest that uplift in Taurides beginning 8 Ma and progressing in stages, reaching a total elevation increase of 2 km. EXP-1 is in good agreement with this estimate in terms of the timing and the amount of uplift between 8–2 Ma. The additional ~1 km of uplift would be related to the tectonic component of slab break-off/tear in the subducting plate under the Cyprus arc^16^. However, the break-off is constrained under the plate convergence zone (south of Taurides) as shown in the tomographic images^19^ therefore such process cannot account for the approximately >1 km broad uplift of the entire Central Anatolian plateau (~650 km) since ~8 Ma. Geodynamic models show that the slab break-off has more confined response in elevating the surface topography (e.g., especially in the zone of slab detachment)^36^ rather than the plate hinterland. The initial phase of subsidence predicted by our drip tectonics models (Fig. 4b) is consistent with elevation estimates derived from back-stripping data from the Tuz Gölü basin^38^. These estimates indicate topographic inversion 3 Ma and total vertical displacement of ~1 km. Though the pattern of inversion from subsidence to uplift can match with the models, the inconsistency in timing may be owing to various factors, such as model assumed rheology and uncertainty in the initial conditions controlling the start of the lithospheric instability, as well as the three dimensional nature of the process that is not captured in model calculations.

Figure 4c shows the modeled crustal thickness variation in the zone of lithospheric removal (~650 km) compared with the seismological (receiver function) estimates^39^. EXP-1 shows the best fit compared to the other models where the crust is ~36 km thick, especially on both sides of the central plateau. The crust is ~6 km thicker in the center of EXP-1 compared to the seismological interpretations, which may be due to the active arc root dripping considered in the models whereas such process may have ended in the Central Anatolia.

The style of crustal deformation concurrent with the inversion to uplift is debated for Central Anatolia. Our preferred drip model (EXP-1) shows a transition from crustal shortening to extension as the lithosphere is removed. The thin viscous sheet calculations predicted in the dynamic model^40^ suggests extension in Central Anatolia, and this is in agreement with the preferred (EXP-1) model predictions. According to geological interpretations^41^ crustal extension has been ongoing since the late Cretaceous accompanied by syn-extensional granitic plutonism and formation of the Tuz Gölü basin. Structural-kinematic analyses along the Tuz Gölü fault led to interpretations that this fault was reactivated as a normal fault ~6 Ma when the regional tectonic regime changed from shortening to N–S to NE–SW extension due to lithospheric scale processes^42^. Normal faulting since the late Pleistocene in southeastern part of Central Anatolia (e.g., Ecemiş fault) has also been inferred by the cosmogenic exposure dating of terrestrial sediments and structural studies^43^. On the other hand, large-scale east-northeast vergent thrusting appears to have continued into the Quaternary as shown in the seismic reflection profile at the Tuz Gölü fault thus disproving the allegations of a switch from shortening to extension (See Figure 5a in ref. ^38^). It may be that the extensional structures in Central Anatolia are controlled by the strike-slip kinematics in the region^44^, as suggested by earthquake focal mechanisms^45^.

Lithospheric foundering processes^46,47^ (e.g., peel away/delamination^9,36,48^ or viscous drip^6,49^) and the participating amount (i.e., mantle lithosphere and/or lower crust) to the removal is dependent on tectonic setting^50^ (i.e., magmatic arcs^51,52^, rift systems^53^, plate convergence zones^54^, intra-plate tectonics^55^, cratons^56^). For example, presumed slab peel away process (e.g., similar to wholesale delamination mechanism) from beneath the Eastern Anatolia is associated with surface uplift (~2 km) and widespread melt production under the entire Eastern Anatolia (from Caucasus to the Bitlis suture zone)^36,57^. On the other hand, more localized and coeval volcanism associated with asthenospheric mantle source (since ~10 Ma) in the north (Galatia) and south (Cappadocia) in Central Anatolia may be more consistent with the viscous drip type removal following to the folded continental arc (hence thickened at depth). These different foundering styles—between the Central and Eastern Anatolia—have also been suggested through analysis of geochemical characteristics of volcanics^58^ and the different potential mantle temperatures^23^. Therefore, a single continental delamination model^59^ that accounts for the uplift and tectonic evolution of both Central and East Anatolia may be questionable, although the mantle driven dynamic topography most likely contributed to the ~1 km uplift of both plateaus^60^. Slab roll-back in conjunction with the small-scale drips under Central Anatolia is plausible mechanism to produce shallow melting inferred by petrological interpretations for the Hasandağ basalts^23^, however, slab retreat does not explain the >1 km of surface uplift at the back-arcs since 10 Ma. The plate hinterlands/back-arcs in these settings are associated with extension/subsidence—e.g., in the Aegean and other Mediterranean basins^2,7,8^, rather than surface uplift, well constrained in Central Anatolia.

It is possible that the initiation of the lithospheric drip ~10 Ma may be triggered or facilitated by the reduced effective viscosity of the sub-arc mantle lithosphere due to the asthenospheric mantle entrainment under the Anatolian plate from the east. Hot mantle intrusion could occur through asthenospheric mantle upwelling by a slab peel away from the accretionary crust in the Eastern Anatolia^36,57,61^. A slab window opening due to the slab break-off^16^ under Cyprus may effectively favor the initiation of the lithospheric instability and following dripping process by hot mantle passage from the south. However, it would presumably occur later (~2 Ma), following the collision between the Eratosthenes Seamount and Cyprus arc^62^, therefore, its influence may relatively be in secondary importance. Future work on identifying more precise timing of the transition from calc-alkaline to alkaline volcanism, structural controls on the basin formations (normal, strike slip, and thrust tectonics), geochemical characteristics of the sub-arc mantle lithosphere (e.g., investigations on the eclogite bearing xenoliths) and their relation to the deep high resolution geophysical images (seismic tomography, receiver functions, MT) will all help us to better understand the style and character of the lithospheric removal.

## Methods

### Numerical technique

The numerical code employed here, SOPALE, uses arbitrary Lagrangian–Eulerian (ALE) finite element techniques to solve for the plane-strain deformation of complex visco-plastic materials^63^. The ALE technique is useful for treating finite deformations, and for tracking boundaries (surface and Moho topography) and internal particles (P-T paths)^8,9,36,50^. The configuration of the model is designed as a general representation for the gravitationally unstable arc root removal from beneath the crust into the mantle.

### Model design and material properties

A broad suite of drip tectonics models that explore a large range of modeling space (e.g., density, activation energy, viscosity, initial size and the width of the drip) are conducted in this work. By showing a representative suite of experiments in the context of orogenic evolution of Central Anatolia (and possibly to other areas where dripping lithosphere has been proposed), we have focused on cases tailored to the region and highlighting the influence of the important parameters. The choice of major model parameter approximations for Central Anatolia are given below (please see Supplementary File 1 for other model parameters).

The wet quartzite rheology used in these models is an approximation for the general lithological properties of the Central Anatolian basins (e.g., Tuz Gölü basin) that are made up of thick layers of Oligo-Miocene conglomerate and Pliocene sandstones^29^. When weak lower crust is inserted in the models (e.g., felsic granulite rheology) the dense layer starts to peel away/delaminate depending on the lateral extent of the weak layer^9^. Based on the same flow law used for the mantle (see Model set-up section), the viscous deformation of the continental crust is controlled by the material parameter (*A* = 1.1 × 10^28^ Pa^−4^ s^−1^), power law exponent (*n* = 4), and the activation energy (*Q* = 223 kJ mol^−1^) based on wet quartzite^64^ (Supplementary Table 1). In addition to the viscous response, the crust is able to deform by frictional plastic yielding in which the Drucker–Prager yeld criterion is used, equivalent to the Coulomb criterion in plane strain, *σ*_y_ = *p *sin *ϕ* + Cc (crust). For the crust, an empirical weakening is imposed with the internal angle of friction varying from *ϕ* = 15−2° dependent on the strain. Here, *ϕ* = 15° is an effective internal angle of friction that implicitly includes the effects of pore fluid pressure *P*_f_ in the crust. The weakening/softening process of the crust represents the increasing fluid pressure through partial melt and fluid infiltration into the Kırşehir arc by subduction processes. This is a regular approach in these types/scales of models (e.g., ref. ^50^) Furthermore, the crustal weakening employed in these models implicitly takes into account the shear zone related deformations (e.g., cataclastic flow, fault gouges)^65^. In these experiments, the isostatic thickening of the crust is also effective in increasing the elevation of the surface topography, even without asthenospheric mantle upwelling. Further, such elevation change is amplified compared to a perfect Airy isostasy most likely because of the strain weakening implemented in the crust. For instance, when comparing the results of EXP-3 against EXP-1 at later stages the crustal thickening of 7 km corresponds to change in elevation from 1 to 2 km in the center of the drip where asthenospheric mantle upwelling does not occur. For a perfect Airy type isostasy, 1 km of surface topography is associated with ~5.6 km of crustal thickening (assuming *ρ*_cont = _2800 kg m^−3^ and *ρ*_m = _3300 kg m^−3^).

A locally pre-existing perturbation (280 km wide and 160 km thick) represents a shortened and thickened Kırşehir arc root at the center of the model that instigates the removal process. Inferences made by geodynamic models suggest that such plate shortening may not only effect the crust and but also the underlying mantle part of the lithosphere (sub-arc lithosphere) which may lead to lithospheric instabilities^6,49,50^. The initial width of the arc (~280 km) is based on the distribution of arc-related granitoids in Central Anatolia, and the 160 km thickness is chosen for 25% thickening, inferred by paleomagnetic restorations^31^. The geometry of the instability is chosen based on available observational constraints for the arc root under Central Anatolia. The duration of the models is kept in the 0–10 My interval because this was when the plateau uplift started to occur and possibly switched from shortening to extension at ~6 Ma.

In these experiments, the reference density of the sub-arc mantle lithosphere (arc root) is initially set to be higher than the underlying asthenospheric mantle. Petrological studies infer the opposite buoyancy conditions especially for older lithospheric plates (e.g., cratons)^47^ due to chemical depletion. However, owing to several geologic factors a higher density of the arc root is suitable for the ~80 my old Central Anatolia (Kırşehir) continental magmatic arc. First, sub-arc mantle lithosphere (arc roots) in thickened/shortened mature arcs, (e.g., Late the Cretaceous arc in Sierra Nevada) may become denser because of refertilization/enrichment due to the entrapment of the melts induced by subduction fluids^66^. The sub-batholitic arc root for various regions is considered to be of high relative density^47,51,67^, as with the Kırşehir arc root. In support of this interpretation, it has been suggested that the Central Anatolian basalts (Cappadocia area) carry components of enriched/refertilized mantle, interacted with slab derived fluids^23^. A second geologic factor for the dense unstable arc root in Central Anatolia is potential eclogitization of the lower crust. Shortening of lower crust (as in the region) can transform gabbroic rocks into eclogitic facies conditions^68^. Subsequently, eclogites in the lower crust can sink into the underlying arc root and promote the instability with other mafic residues^69^. Though the mechanism is plausible for Central Anatolia, the evidence for the presence of eclogite bearing xenoliths has not been yet been substantiated.

The effects of varying viscosities of the dripping arc root are tested and an alternative model with 10 times higher viscosity (ranging from *μ* = 5.10^20^‒5.10^22 ^Pa s for the same strain rates as the preferred experiment EXP-1) is shown in Supplementary Fig. 2. The EXP-1 is associated with lower arc root viscosity which controls the initiation of drip which started at ~10 Ma. This timing is and the evolution of surface tectonics is consistent with the geological evolution of Anatolia.

The numerical (width) and (depth) resolution is 201 × 101 Eulerian nodes and 601 × 301 Lagrangian nodes. Half of the Eulerian and Lagrangian elements are concentrated in the top 160 km in order to enhance resolution in the lithosphere. The model has a free top surface, allowing topography to develop as the model evolves. The mechanical boundary conditions at the other three sides are defined by zero tangential stress and normal velocity (e.g., “free slip”). We have extended the depth of the solution space into the lower mantle so that the sinking mantle lithosphere material moves away from the lithosphere. The initial geotherm for the experiments is laterally uniform and is defined by a surface temperature of 25 °C, an increase to 550 °C at the Moho, an increase to 1350 °C at the base of the mantle lithosphere, and an increase to 1525 °C at the bottom of the model. We tested the influence of Moho temperature (350–750 °C) in a series of models and the general model predictions discussed here (e.g., surface elevation, crustal thickness) are only minorly affected by this.

The surface and bottom temperatures are held constant throughout the experiments and the heat flux across the side boundaries is zero. The initial temperature profile is the same in all experiments. Thermal properties (thermal conductivity *k* = 2.25 W m^−1^ K^−1^, heat capacity *c*_p = _1250 J kg^−1^ K^−1^) are the same for all materials and we ignore radioactive heat production and shear heating in the model. In this work we do not take into account explicitly petrological processes of decompression and/or hydrated mantle melting.

### Data availability

The data that support the findings of this study are available from the corresponding author upon reasonable request.

## Electronic supplementary material

Supplementary Information
