# Supplementary material for: Drip tectonics and the enigmatic uplift of the Central Anatolian Plateau
Source: Nat Commun. 2017 Nov 16;8:1538. doi: 10.1038/s41467-017-01611-3 (PMC5688165; doi:10.1038/s41467-017-01611-3)
Supplement: Supplementary file 1 — Supplementary Information [file 41467_2017_1611_MOESM1_ESM.pdf]

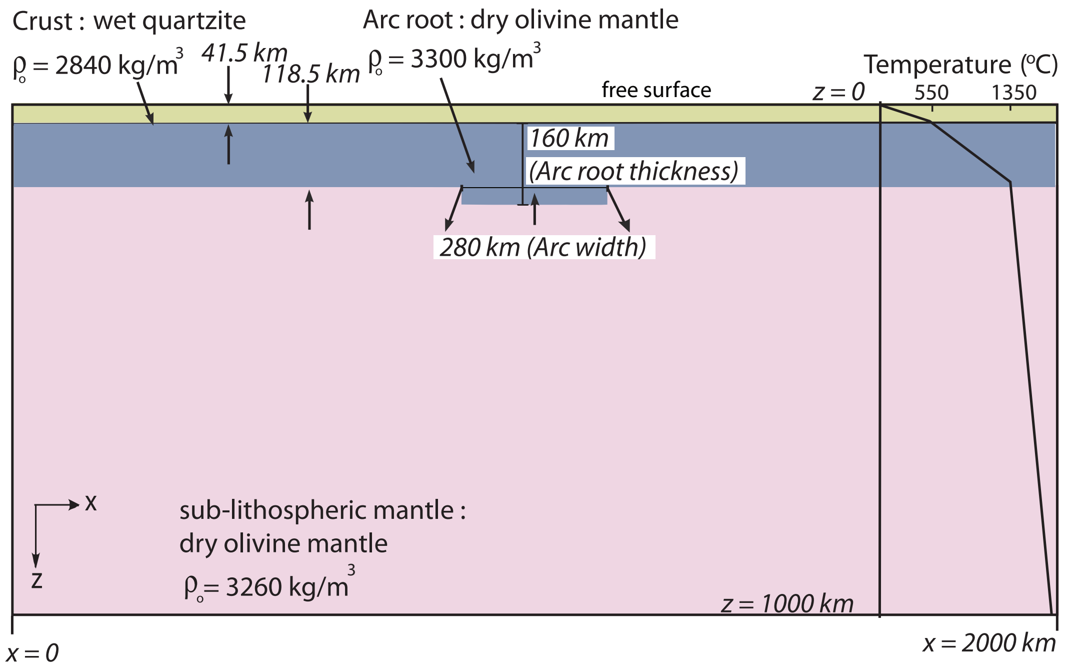

Supplementary Figure 1: Illustration of the model geometry, set-up, material properties, and density field for the preferred drip model experiment (EXP-1). In the models, the 160 km thick lithosphere is made up of 41.5 km thick crust (ρ_o= 2840 kg m^{-3}, yellow) and 118.5 km thick mantle lithosphere (ρ_o = 3300 kg m^{-3}, blue) overlying an sub-lithospheric mantle region (ρ_o = 3260 kg m^{-3}, pink). The width (280 km) and the thickness (160 km) of the arc root instability are based on geological, Görür et al [1] and paleomagnetic studies on the Late Cretecaus granitoids of Central Anatolia by Lefebvre et al. [2]. Initial crustal thickness beneath the arc (41.5 km) is based on the estimates from P-wave derived seismic Moho under the Costa Rica- Central American arc ~ 70 Myrs old Gazel et al. [3] and the approximate crustal thickness under Kohistan and Talkeetna arcs Jagoutz and Behn [4]. The 160 km thick lithosphere over the model domain is based on the Late Cretaceous Sierra Nevada arc as base of the sub-batholith mantle lithosphere (e.g., including peridodite and mantle wedge) is at ~ 140 km depth inferred by Saleeby et al [5], as an approximation to the late Cretaceous Central Anatolia-Kırşehir arc. Similarly, a 120 to 150 km boundary for the base of the lithosphere in the Cordilleran arc system (e.g., Altiplano) was suggested by Heit et al [6].

	Parameter	Continental Crust	Mantle lithosphere (Arc root and elsewhere in the model)	Sub-lithospheric mantle (Asthenosphere)
A	Viscosity parameter	1.1 x 10 ^{28}Pa^{-4} s^{-1}	10^{-38} Pa^{-n} s^{-1}	4.89×10^{-17}Pa$^{-3.5}$ s^{-1}
n	Power exponent	4.0	3.5	3.5
Q	Activation energy	223 kJ mol^{-1}	0	535 kJ mol^{-1}
ϕ	Effective Internal Angle of friction	15°-2°	0	0
ρ_o	Reference Density	2840 kg m^{-3}	3300 kg m^{-3}	3260 kg m^{-3}
σ_y	Plastic yield stress	1 MPa	0	0
α	Coefficient of thermal expansion	2.0 X 10^{-5} K^{-1}	2.0 X 10^{-5} K^{-1}	2.0 X 10^{-5} K^{-1}

Supplementary Table 1: **Rheological parameters for the preferred numerical experiment (EXP-1)**

Please see methods section for the references of other model parameters (e.g density, internal angle of friction). Note that the mantle lithosphere in EXP-1, EXP-3 and EXP -4 is associated with temperature-independent rheology (Q = 0) but the rheological parameters for the underlying sub-lithospheric mantle (dry olivine mantle) is based on the experimental results from Hirth and Kohlstedt [7] (A = 4.89×10^{-17}Pa$^{-3.5}$ s^{-1}, 535 kJ mol^{-1}). For EXP-2 with temperature dependent mantle lithosphere rheology all mantle layers are based on the Hirth and Kohlstedt [7]. The material parameters for the wet quartzite crust are from Gleason and Tullis [8].

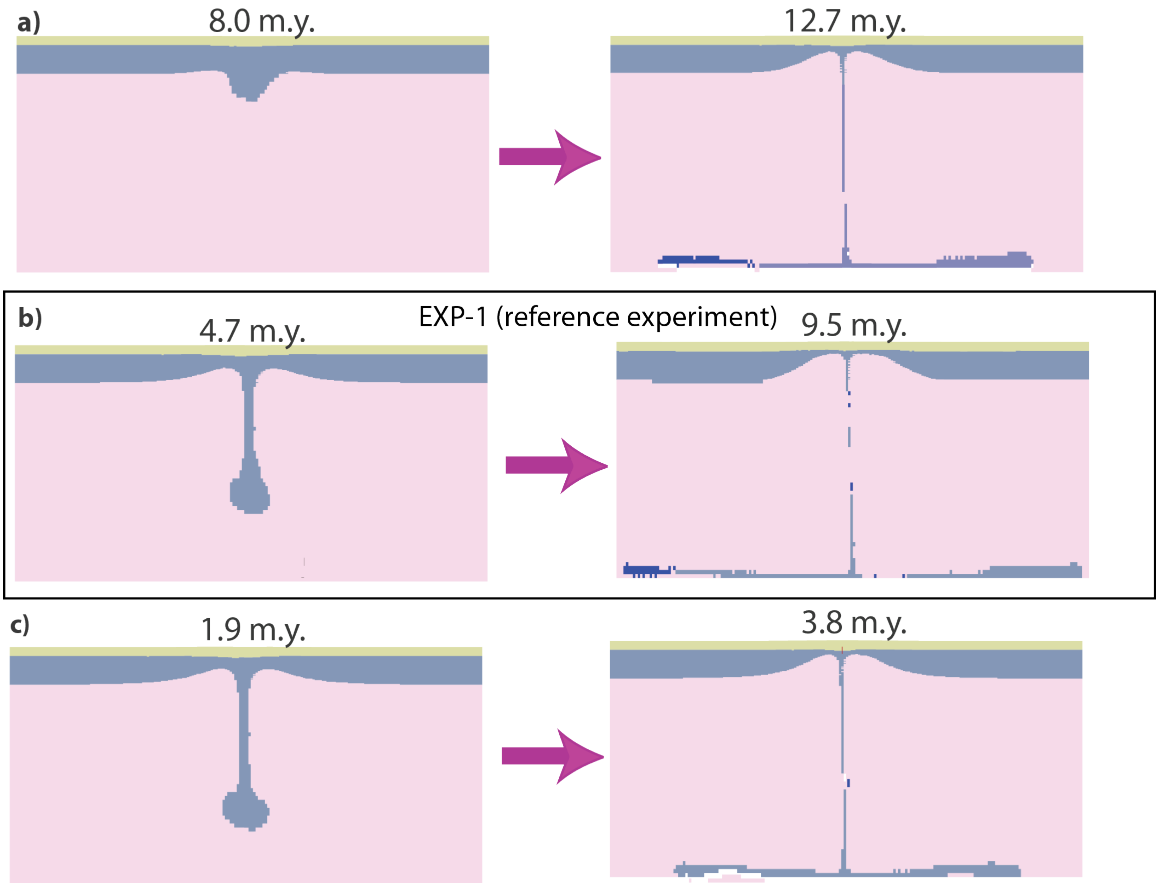

Supplementary Figure 2: Comparison of the geodynamic evolution between dripping lithosphere experiments in which density difference between the sub-arc mantle lithosphere (arc root, blue region) is investigated *(*$\Delta\rho = \rho_{arcroot} - \rho_{asthenosphere}$*)*. The reference experiment (EXP-1) with $\Delta\rho = 40$ kg m^{-3} is shown in; b) middle and the one a) above is $\Delta\rho = 30$ kg m^{-3}, whereas the the one below; c) shows $\Delta\rho = 60$ kg m^{-3}. All other model parameters are kept same. Note that such a density difference in EXP-1 is in good agreement with the dynamics of the lithospheric removal and the observed surface uplift of 1 km that occured 8-2 Ma in Central Anatolia. a) when the density difference is decreased to 30 kg m^{-3} the process slows down by ~ 4.5 Myrs and c) when it is increased to 60 kg m^{-3} the removal occurs very rapidly and surface uplift occurs in 3-4 Myrs. Our choice of density difference is also comparable to density difference estimates between the arc root lithosphere and the underlying less dense mantle inferred by Jull and Kelemen [9].

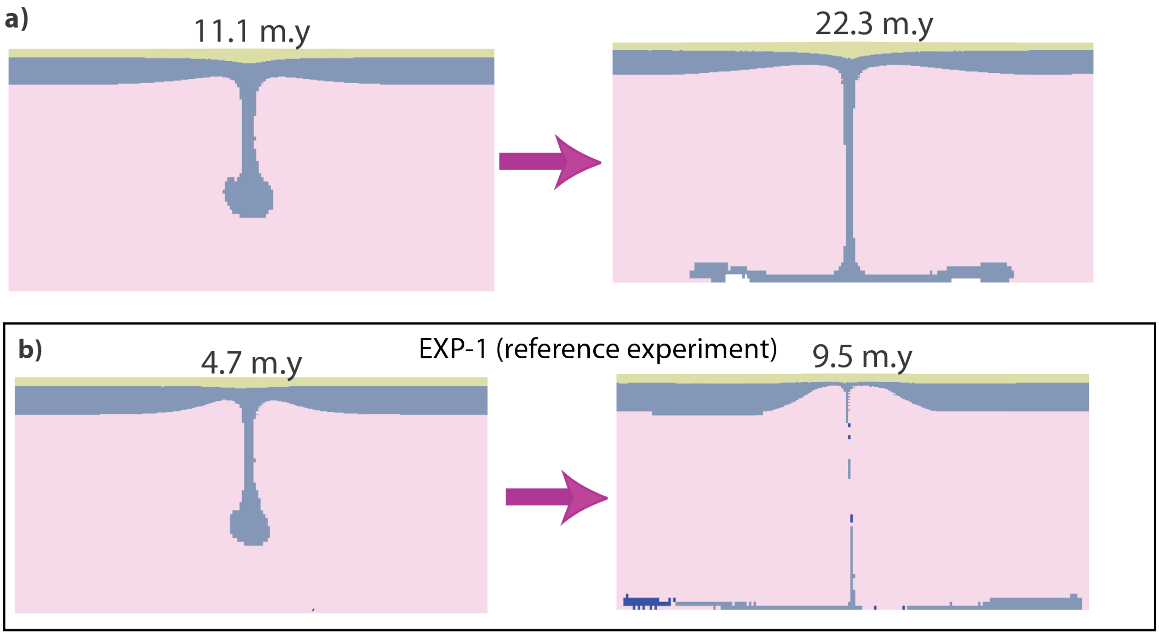

Supplementary Figure 3: Comparison of the geodynamic evolution between dripping lithosphere experiments in which viscosity (μ) of the sub-arc mantle lithosphere (arc root, blue region) is modified a) The viscosity ranges from $\mu = 5.10^{20}$ - 5.10^{22} Pa s for the same strain rate range used in the preferred model (e.g strain rates 10^{-12} to 10^{-17} s^{-1}) b) The preferred experiment (EXP-1) is shown where the viscosity variation ranges between 5×10^{19} - 5×10^{22} Pa s. Note that the increasing viscosity of the arc root delays the dripping process significantly ~ > 10 Myrs, as well the restriction of the amount of proceeding mantle upwelling under the crust.

Supplementary references

[1] Görür, N., Tüysüz, O., & Sengör, A.M.C. Tectonic Evolution of the Central Anatolian Basins. *International Geology Review* **40,** 831-850, (1998).

[2] Lefebvre, C., M. J. M. Meijers, Kaymakci, N., Peynircioğlu, A., Langereis, C.A., & van Hinsbergen, D. J. J., Reconstructing the geometry of central Anatolia during the late Cretaceous: Large-scale Cenozoic rotations and deformation between the Pontid es and Taurides. *Earth and Planetary Science Letters* **366**, 83–98, (2013)

[3] Gazel, E., Hayes, J.L., Hoernle, K., Kelemen, P., Everson, E., Holbrook, S.W., Haff, F., van Den Bogaard P., Vance, E.A., Chu, S., Calvert, A.J., Carr., J.M., & Yogodzinski, G.M. Continental crust generated in oceanics arcs. *Nature Geoscience* **8** 321-327, doi: 10.1038/NGEO2392, (2015).

[4] Jagoutz, O., & Behn, M.D. Foundering of lower island-arc crust as an explanation for the origin of the continental Moho. *Nature* **504**:131-134 (2013).

[5] Saleeby, J., Ducea, M, & Clemens-Knott, D. Production and loss of high-density batholithic root, southern Sierra Nevada region. *Tectonics* **22**, 6, 1-24, doi:10.1029/2002TC001374 (2003).

[6] Heit, B., Sodoudi, F., Yuan, X., Bianchi, M., and Kind, R. An S-receiver function analysis of the lithospheric structure in South America. *Geophysical Research Letters* **34**, L14307, doi: 10.1029/2007GL030317, (2007).

[7] Hirth, G., & Kohlstedt, D. L. Water in the oceanic upper mantle: Implications for rheology, melt extraction and the evolution of the lithosphere. *Earth and Planetary Science Letters* **144**, 93–108, doi: 10.1016/0012-821X (96)00154-9, (1996).

[8] Gleason, G. C., & Tullis, J. A flow law for dislocation creep of quartz aggregates determined with the molten salt cell. *Tectonophysics* **247**, 1-23, doi: 10/1016/0040-1951(95)00011-B, (1995).

[9] Jull, M., & Kelemen, P. B. On the conditions for lower crustal convective instability. *Journal of Geophysical Research* **106,** 6423–6446 (2001).
